# Expected professional competencies articulated in learning outcomes for novice nursing students in early nursing home placements: a document analysis

**DOI:** 10.1186/s12909-026-10048-6

**Published:** 2026-07-30

**Authors:** Berit Mosseng Sjølie, Mette Kristin Ragnhildstveit Sætra, Monika Ravik, Hanne Maria Sørensen Bingen, Bjørg Christiansen, Marianne Trygg Solberg

**Affiliations:** 1 Faculty of Nursing and Health Sciences, Nord University, Campus Vesterålen, Storgata 105, Leknes, 8370 Norway; 2 Department of Health and Nursing Science, University of Agder, Campus Grimstad, Jon Lilletuns vei 9, Grimstad, 4879 Norway; 3 Faculty of Health and Social Sciences, Department of Nursing and Health Sciences, University of South-Eastern Norway, Campus Porsgrunn, Kjølnes Ring 56, Porsgrunn, 3918 Norway; 4 Lovisenberg Diaconal University College, Lovisenberggata 15B, Oslo, 0456 Norway; 5 Centre for Senior Citizen Staff, OsloMet – Oslo Metropolitan University, Pilestredet 46, Oslo, 0166 Norway

**Keywords:** Clinical placement, Professional competence, Nursing education, Health professions education, Learning outcomes, Taxonomy

## Abstract

**Background:**

Clinical placements are central to the development of professional nursing competence, particularly in the first year of undergraduate education. In many countries, nursing students begin clinical education in nursing homes, where learning opportunities may vary and structured supervision can be limited. Clearly formulated learning outcomes are therefore essential for guiding learning, teaching and assessment. Despite extensive use of competence‑based frameworks and learning taxonomies, limited attention has been given to how professional competencies are articulated and taxonomically positioned in learning outcomes for first‑year nursing home placements.

**Aim:**

To identify the professional competence areas articulated in learning outcomes for first‑year nursing students’ nursing home placements, and to examine how these outcomes are distributed across cognitive, affective and psychomotor learning domains and their associated taxonomic levels.

**Methods:**

Learning outcomes formulated in subject plans for first‑year nursing home placements were analysed using a deductive document analysis approach. Outcomes were coded to predefined competence areas and mapped to cognitive, affective or psychomotor learning domains. Each outcome was classified as representing a low, intermediate or high taxonomic level. Frequency counts were used to identify patterns across institutions.

**Findings:**

All five professional competence areas were represented; however, their distribution was uneven. Learning outcomes related to individualised care, particularly ethical values and communication, were most prevalent and spanned several taxonomic levels. In contrast, participatory decision‑making, cultural humility, evidence appraisal and higher‑order critical thinking were less frequently articulated and seldom positioned at high taxonomic levels. Across competence areas, learning outcomes were predominantly formulated at low and intermediate levels.

**Conclusion:**

Learning outcomes for first‑year nursing home placements prioritise foundational competencies and safe participation in clinical practice but provide limited opportunities for early development of advanced clinical reasoning and evaluative judgement. Strengthening taxonomic progression in early learning outcomes may better support professional competence development from the outset of nursing education. These findings have implications for curriculum design, assessment alignment and clinical supervision in early health professions education.

**Supplementary Information:**

The online version contains supplementary material available at https://doi.org/10.1186/s12909-026-10048-6.

## Introduction

Learning and development of professional nursing competence during clinical placements is crucial to nursing education, equipping students for future professional roles and the complex demands of real-world healthcare [1–3]. In many countries, first-year nursing students begin their clinical education in nursing home settings, which offer valuable exposure to complex patient needs but also present substantial pedagogical challenges [4, 5]. Studies show that students in early nursing home placements often encounter insufficient learning situations and limited structured support, which underscores the importance of clearly formulated learning outcomes that can guide learning and supervision [6]. Similar challenges have also been identified across other primary healthcare placement settings, where variation in supervisory preparation and support may influence learning quality [5]. However, the present study focuses specifically on learning outcomes formulated for first-year nursing home placements. Together, these findings highlight the critical role of clear, structured learning outcomes in ensuring consistent and supportive learning environments.

Learning outcomes describe the intended instructional results by combining subject matter content (noun phrase) with a cognitive process (verb phrase) [7]. In line with Anderson et al. [8], the term “learning outcomes” is used throughout to denote the intended student learning results, related terms such as curriculum standards and learning goals are treated synonymously. In Norway, the Norwegian National Qualifications Framework for Lifelong Learning (NQF) operationalises competence primarily in terms of observable behaviours. This differs from international qualification frameworks, such as the European Qualifications Framework (EQF), which typically organise learning outcomes into the domains of knowledge, skills and competence [9]. These differing approaches indicate that the competence underpinning learning outcomes is not uniformly understood or applied across frameworks. Different qualification framework traditions may therefore influence how learning outcomes are formulated and interpreted. Consequently, comparisons of learning outcomes across institutions and national contexts should be made with caution, as apparent differences may partly reflect framework-related variations rather than substantive differences in educational expectations.

Professional nursing competence is central but ambiguously defined, and the lack of consensus complicates efforts to standardise education and practice, including the formulation of learning outcomes for early clinical placements such as first-year nursing home placements [3, 10, 11]. This study adopts the World Health Organization’s [12] definition of professional nursing competence, which conceptualises competency as a broad composite framework reflecting knowledge, skills and attitudes, and psychosocial and psychomotor elements. Later WHO (World Health Organization) frameworks operationalise these components through observable professional behaviours. To provide a comprehensive framework for nursing education and ensure consistency across clinical placements, a scoping review by Purabdollah et al. [3] identified five overarching competence areas: 1) Individualised care, 2) Evidence-based nursing care, 3) Professional nursing process, 4) Nursing administration and 5) Work readiness and professional development. Each of these areas includes several subdomains that together form a comprehensive competence framework that help to structure learning outcomes and standardise educational content across clinical placements [3].

## Background

In Norway, as internationally, bachelor’s nursing programmes use learning outcomes to define what students are expected to do during clinical placements [13, 14]. To improve clarity and alignment on teaching and assessment, these learning outcomes are often organised into competence areas [3]. However, systematic grouping and taxonomic mapping of learning outcomes specifically for first-year clinical placement in nursing home settings remain unexplored [3, 15–18]. This knowledge gap is particularly relevant in nursing home placements, which constitute the first clinical placement experience for many Norwegian nursing students and therefore play an important role in shaping early professional development.

Competence areas provide a systematic framework for teaching and assessment in first-year nursing home placements, enabling students to develop a well-rounded skill set aligned with the demands of professional nursing practice [3]. Further, linking learning outcomes to defined competence areas allows educators and Registered Nurse mentors to align teaching strategies and evaluation methods with the complexities of clinical placement, better preparing students to meet both educational and healthcare system requirements [1].

Effective evaluation of students in clinical practice requires that learning outcomes explicitly reflect the competence demands of the clinical tasks that are expected of nursing students during their clinical placement studies [19]. Ensuring that teaching activities and assessment strategies are aligned with learning outcomes at appropriate taxonomic levels is therefore crucial, as such alignment supports coherent progression towards higher-order clinical reasoning and competent performance [8, 20–22].

This emphasis on alignment is best interpreted in light of the three domains of learning and their associated taxonomies. Across educational theory, three domains of learning are commonly recognised: the cognitive (knowledge), the affective (attitudes and values) and the psychomotor (skills). Drawing on Bloom’s taxonomy and its extensions, Pettersen [23] emphasises that each domain is structured as a hierarchy progressing from simple to increasingly complex levels of mastery.

The cognitive domain relates to mental processes such as knowledge, understanding and problem-solving. This is the domain that was originally developed by Bloom [24] and later revised by Anderson et al. [8]. The revision transformed the categories from nouns into action verbs. The affective domain, developed by Krathwohl et al. [25], describes how learners develop attitudes, values and emotional engagement, progressing from basic receptiveness to stimuli, through participation and valuing, towards the internalisation of values that guide behaviour. According to Pettersen [23], the psychomotor domain encompasses practical skills and has evolved from three traditions: Dave (1970/1975), Simpson [26] and Harrow [27]. These models are described as outlining a developmental progression from imitation and basic movements to precision, complex performance and creative action [23].

The taxonomic levels collectively provide a structured foundation for guiding students’ intellectual development and for ensuring that learning activities and assessments are sufficiently challenging [8]. All domains of learning that constitute the competence must receive adequate attention, and the content and learning processes within each dimension must gradually incorporate learning outcomes at higher taxonomic levels [21, 23].

Gaining a clearer understanding of how students develop professional competence during placements is therefore essential. It allows educators and clinical staff to gauge the impact of the learning environment and offers valuable guidance for organising placements and prioritising the skills that are most critical for students’ professional growth [3, 15–18]. However, the literature has yet to thoroughly examine the specific professional competencies expressed in the learning outcome descriptions for clinical placement studies in nursing education, and the expectations embedded in these formulations [28]. Therefore, this study aimed to identify the professional competencies articulated in learning outcomes for first-year nursing students in nursing home placements, and to describe how these outcomes are distributed across cognitive, affective and psychomotor domains and their taxonomic levels. The following research questions were developed:

- Research questions 1: Which competence areas are articulated in the learning outcomes for first-year students’ nursing home placements?
- Research questions 2: How are the learning outcomes distributed across the cognitive, affective and psychomotor domains and their associated taxonomic levels?

### Theoretical perspectives

The theoretical framework for this study integrates the five competence areas identified by Purabdollah et al. [3], which encompass the essential skills and knowledge required for effective nursing practice. These areas offer a structured foundation for nursing education, ensuring students are equipped to meet the diverse challenges of clinical placements.

#### The competence areas by Purabdollah et al. [3]

*Individualised care* ensures that nursing care is tailored to meet the unique needs of each patient, fostering patient-centred care and improving health outcomes. This involves respecting ethical values and codes, maintaining dignity and integrity, and practicing therapeutic communication that engages patients and families in participatory decision-making. It also includes promoting patient-centred care, ensuring that care aligns with individual preferences and needs, followed by demonstrating cultural humility, adapting care to diverse cultural contexts and supporting a holistic approach to well-being.

*Evidence-based nursing care* emphasises the integration of research and best practices into clinical decision-making, ensuring high-quality, effective and up-to-date patient care. This competency involves knowledge acquisition in biomedical sciences, nursing theory and research literacy, combined with the ability for critical appraisal and implementation of applicable evidence to provide safe, cost-effective and scientifically grounded care.

*Professional nursing practice* equips nurses with the ability to assess, plan, implement and evaluate care systematically, ensuring a structured and effective approach to healthcare delivery. This competency relies on critical thinking skills at three levels: general (problem-solving and cognitive ability), specific (clinical reasoning and sound judgement) and professional (applying the nursing process through assessment, diagnosis, planning, intervention and evaluation).

*Nursing administration* focuses on leadership, organisation and resource management, which are crucial for coordinating care and maintaining the efficiency of healthcare systems. It includes management and leadership skills such as prioritisation, delegation, teamwork and interprofessional collaboration, as well as proficiency in informatics and documentation to ensure accurate records and effective use of digital tools in clinical practice.

*Work readiness and professional development* prepares nurses to adapt to the demands of the healthcare environment, fostering lifelong learning and ensuring that they remain competent and resilient in their roles. This competency includes personal characteristics such as self-regulation, adaptability, stress management and professionalism; awareness of legality, including legal standards and professional responsibilities; proficiency in safety procedural skills for delivering safe care; engagement in preventive health services, such as health promotion and risk prevention; and development of mentoring competency, which involves teaching, coaching, role modelling and supporting career growth.

## Design and methods

This study is part of the Norwegian Network for Educational Research in Nursing, which involves all 13 educational institutions with formal representatives. The overall study aims to analyse core curriculum documents related to pedagogical and nursing aspects in placement studies. Institutional leaders consented to provide access, and representatives collected documents to create a digital database for project data retrieval.

For this study, a qualitative descriptive design employing descriptive document analysis methodology was utilised. A descriptive research design is appropriate when the aim is to systematically describe and understand the characteristics of a particular phenomenon [29]. As a qualitative research approach, document analysis entails a methodological examination and evaluation of documents to gain a deep understanding and empirical insights [30].

### Research context

Nursing homes play a vital role in healthcare, providing specialised care for the ageing population and vulnerable persons [31, 32]. These settings expose nursing students to chronic conditions such as diabetes, hypertension and cardiovascular diseases [33], which require ongoing care, patient education and support for self-management [34]. In Norway, nursing homes serve as the first clinical placement for first-year nursing students, offering a dynamic learning environment [4]. Here, students should learn to develop key competencies, including caring for older adults, managing complex health needs, and addressing ethical and professional challenges, thereby forming a foundation for their nursing education [4, 14, 35].

### Data collection

The empirical material used in this study comprised formal documents from all nursing education programmes in Norway for the academic year 2022/2023 (autumn 2022–summer 2023) (*N* = 13). The empirical material comprised formal curriculum documents from all Norwegian bachelor’s nursing programmes. Only learning outcomes formulated in subject plans for first‑year nursing home placements were included in the analytical dataset, and study plans were used to provide contextual and structural information. We focused on subject plans for first‑year clinical placements in nursing homes. The material collected for the study was extensive, and to comply with the article format, only selected excerpts from the data material are presented to illustrate the findings.

### Ethical considerations

This study analysed formal curriculum documents from Norwegian bachelor’s nursing programmes. All documents were either publicly accessible or provided with institutional permission and did not contain personal or sensitive information, thereby minimising risks related to confidentiality and privacy. Formal leaders of all participating educational institutions were informed about the project and consented to providing access to the relevant documents.

Only learning outcomes formulated in subject plans for first‑year nursing home placements were included in the analysis, whilst study plans were used solely to provide contextual and structural information.

Although the material was institutional in nature, care was taken to ensure respectful and fair treatment of the educational institutions involved. To reduce the risk of unintended evaluation or attribution, each institution was assigned a numerical identifier, and no identifying information was linked to the findings.

Throughout the analysis, the researchers adhered to principles of neutrality and objectivity, ensuring that the documents were represented accurately and without bias. These ethical considerations align with established guidelines for qualitative research, and support transparent and responsible handling of institutional data. Ethical approval was not required, as the study analysed non‑sensitive institutional and publicly available documents in line with national guidelines.

### Analysis

**Fig. 1:**
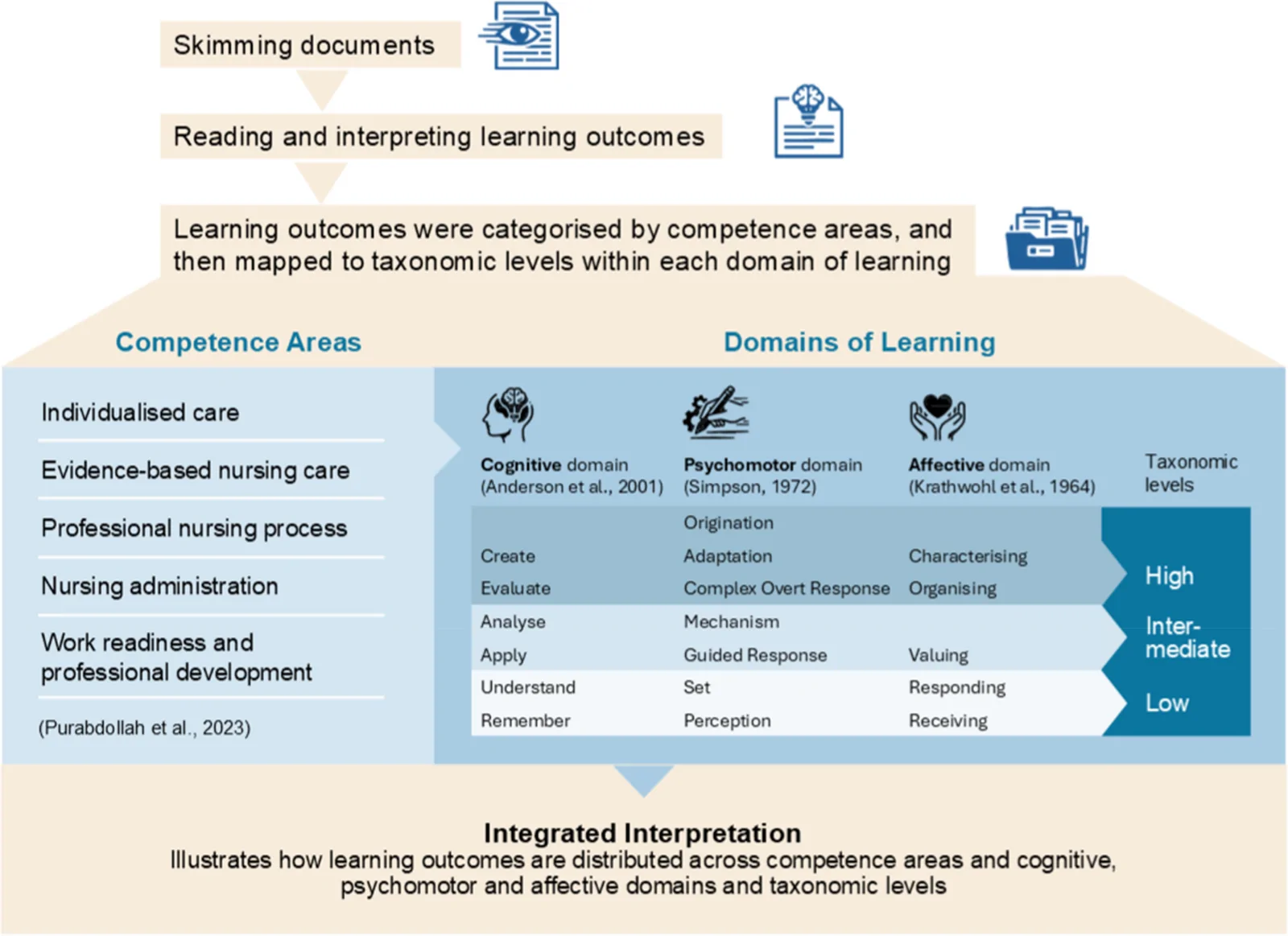
Analytical process for categorising and coding learning outcomes

A deductive approach was applied to assess how well the document content aligned with predefined categories [30]. Following Bowen’s [30] guidelines, the analysis began by skimming the documents to obtain an overview, and continued with close reading and interpretation to understand the meaning of the material. We first coded the learning outcomes to the core competencies described by Purabdollah et al. [3], and then coded each outcome to the taxonomic levels within the relevant learning domain, the cognitive domain [8], the affective domain [25] and the psychomotor domain [26]. Classification was informed by the original descriptions of the cognitive, affective and psychomotor taxonomies rather than by the application of predefined verb lists. Coding was based on the overall competence and level of performance reflected in each learning outcome. Learning outcomes emphasising knowledge, understanding or reasoning were assigned to the cognitive domain, those primarily addressing values, attitudes or professional dispositions were assigned to the affective domain, and those describing practical performance were assigned to the psychomotor domain. Further, we operationalised the hierarchy into three levels (low, medium and high) to capture more nuanced variations in the complexity of the learning outcomes (see Fig. 1). Illustrative examples of the classification process, including interpretation, assignment to competence areas, learning domains and taxonomic levels, are provided in Supplementary File 1. Building on distinctions between lower order and higher order taxonomic levels is recommended by Adams [36].

Our research team consists of six authors (BMS, MKRS, MR, HMB, BC and MTS), with a core group of four researchers (BMS, MKRS, MR and MTS) handling the analysis process. Initially, the core group individually reviewed the material to obtain a comprehensive understanding, and collaboratively distributed the subject plans among themselves. Each learning outcome was systematically reviewed and assigned to the predefined sub competence area defined by Purabdollah et al. [3] (Table 1).

**Table 1:** Competence area defined by Purabdollah et al. [3]

Main competence area	Sub competence area
Individualised care	Value and ethical codes
	Therapeutic communication
	Participatory decision-making
	Patient-centred care
	Cultural humility
Evidence-based nursing care	Knowledge acquisition
	Critical appraisal of evidence and implementing of applicable evidence
Professional nursing process	General critical thinking
	Specific critical thinking
	Professional critical thinking
Nursing administration	Management and leadership
	Informatics and documentation
Work readiness and professional development	Personal characteristics
	Legality
	Safety procedural skills
	Preventative health services
	Mentoring competency

The descriptions of learning outcomes as formulated in the subject plans were treated as units of meaning. Each unit was assigned to at least one sub competence area and represents the analysis unit. BMS reviewed the initial placements, and any ambiguous or multiply assigned learning outcomes were discussed and resolved by BMS, MKRS and MTS until consensus was reached. Some learning outcomes could reasonably be interpreted within more than one learning domain. In such cases, classification was guided by the primary educational intention reflected in the learning outcome. Ambiguous cases were discussed among the researchers and resolved through consensus. Finally, BMS and MTS reviewed the categorisation matrix and adjusted learning outcome descriptions where necessary to ensure consistency across categories.

Following the categorisation (see Table 1), each learning outcome was mapped to the taxonomic levels (low, intermediate and high) within its learning domain (cognitive, affective or psychomotor), and aggregated into its respective competence areas, to enable comparison of cognitive complexity within each competence area. This synthesis summarises how learning outcomes align across competence areas and taxonomic levels, highlighting distributions, overlaps and potential gaps. The supplementary file illustrates this integrated and comprehensive interpretation.

Learning outcomes in each competence area were further counted to provide an overview of how subject plans addressed various aspects of competence areas. Counts were also produced for each taxonomic level (collapsed into low, medium and high), ensuring that every occurrence at each level was recorded within each competence area. In this analysis, counting was used as an analytical strategy to enhance transparency and strengthen the rigour and interpretability of the qualitative findings [37]. In qualitative research, counting does not reduce data to numbers; rather, it helps identify patterns, frequencies and areas of variability [37]. Here, counts were used to show how often each competence area was represented in the learning outcomes that were presented in the subject plans, and to indicate where gaps or underrepresented areas might exist.

By counting the frequency with which competence areas appeared in the learning outcomes, the analysis could highlight not only dominant competence areas but also underrepresented ones, thereby providing a more nuanced understanding of how competence areas were emphasised in the subject plans. Sandelowski [37] also argues that qualitative counting can promote reflexivity, as it encourages researchers to critically examine which elements of the data receive emphasis and how analytical attention is distributed across datasets.

### Trustworthiness

The authors’ extensive background in education and curriculum design supports the credibility of these findings. At the same time, we recognized that our professional backgrounds, personal values, and prior knowledge of nursing education could influence how the document data were interpreted. To address this, the first (BMS) and second (MKRS) authors engaged in ongoing reflective discussions throughout the research process, documenting assumptions and critically examining their potential impact on the analysis.

All six authors contributed to reviewing the article by reading and providing feedback, while the core group focused on the detailed analysis and development of the findings. The researchers were aware that the aim of this study was not to assess the quality of the educational documents but rather to investigate how learning outcome descriptions were articulated in relation to the study’s aim and research questions. We ensured confirmability by fairly representing the document data. To maintain consistency and dependability, the researchers collaborated to resolve differences about what to include in the analysis, and consensus on the results was ultimately achieved. Reflexivity and consensus were established through multiple Zoom meetings throughout the process. To guarantee the accuracy of the final empirical material, the data and coded selections were reread and reviewed in detail.

## Results

The results first present the number and distribution of learning outcome (LO) occurrences in subject plans for first-year nursing home placements across the 13 higher education institutions (HEIs) included in this study (Table 2). Additionally, Table 2 highlights the learning outcomes categorised by competence areas and taxonomic levels.

**Table 2:** Overview of learning outcomes (*N* = 254) in subject plans: distribution and taxonomy levels

Main competence area	Sub competence area	Number of HEIs^1^ that include or do not include LO^2^ (*N* = 13)		Total number of LOs categorised under the sub competence area	Number of LO occurrence (min)	Number of LO occurrence (max)	Taxonomy level		
		Include	Not include				Low	Inter-mediate	High
Individualised care	Value and ethical codes	13	0	28	1	4	7	13	8
	Therapeutic communication	13	0	22	1	4	3	11	8
	Participatory decision-making	7	6	9	0	2	0	4	5
	Patient-centred care	8	5	13	0	3	6	7	0
	Cultural humility	6	7	8	0	2	3	5	0
Total number of LOs categorised under the main competence area 80							19	40	21
Evidence-based nursing care	Knowledge acquisition	11	2	35	0	7	13	4	18
	Critical appraisal of evidence and implementing of applicable evidence	7	6	13	0	3	5	6	2
Total number of LOs categorised under the main competence area 48							18	10	20
Professional nursing process	General critical thinking	3	10	3	0	1	0	3	0
	Specific critical thinking	7	6	16	0	7	3	13	0
	Professional critical thinking	11	2	17	0	3	0	12	5
Total number of LOs categorised under the main competence area 36							3	28	5
Nursing administration	Management and leadership	8	5	22	0	6	16	6	0
	Informatics and documentation	9	4	11	0	3	2	9	0
Total number of LOs categorised under the main competence area 33							18	15	0
Work readiness and professional development	Personal characteristics	9	4	16	0	4	0	16	0
	Legality	8	5	8	0	1	3	5	0
	Safety procedural skills	11	2	27	0	3	0	18	9
	Preventative health services	5	8	5	0	1	1	2	2
	Mentoring competency	1	12	1	0	1	0	1	0
Total number of LOs categorised under the main competence area 57							4	42	11

^1^*HEIs* Higher Education Institutions

^2^*LO* Learning Outcome

The following presentation highlights selected data from Table 2, showing the distribution of learning outcomes in subject plans for first-year nursing home placements across the 13 higher education institutions (HEI 1–HEI 13), across both main and sub competence areas and according to taxonomy level.

### The main competence area ‘individualised care’

A total of 80 instances of learning outcomes related to the main competence areas of *Individualised Care* were identified. Regarding the sub competence areas *Value and Ethical Codes* and *Therapeutic Communication*, learning outcomes were found at all institutions, mentioned between one and four times for both competence areas. *Participatory Decision-Making*, *Patient-Centred Care* and *Cultural Humility* had a lower number of learning outcomes, with either no instances or up to two mentions.

Within the sub competence areas *Value and Ethical Codes* and *Therapeutic Communication*, which contained the highest number of learning outcomes in this main competence area, the distribution spans evenly across low, intermediate and high levels of Bloom’s taxonomy. In the sub competence areas *Patient-Centred Care* and *Cultural Humility*, the learning outcomes were distributed across the low and intermediate levels.

Example learning outcomes:

- *Has knowledge of the professional ethical guidelines for nurses* (HEI 3). Mapped to: Low level, cognitive domain.
- *Plans and carries out communication and interaction with patients and next of kin based on respect, participation and integrity* (HEI 5). Mapped to: Intermediate level, psychomotor domain.
- *Applies ethical principles, professional ethical guidelines and relevant legislation that reflect professional, responsible and safe nursing practice* (HEI 4). Mapped to: High level, affective domain.

### The main competence area ‘evidence-based nursing care’

A total of 48 instances of learning outcomes related to the main competence area *Evidence-Based Nursing Care* were identified. Within the sub competence area *Knowledge Acquisition*, learning outcomes were present at most institutions, with frequency varying across higher education institutions. In contrast, *Critical Appraisal* and *Implementation of Applicable Evidence* were less well represented and absent at several institutions in first-year practice placements.

Within *Knowledge Acquisition*, taxonomy levels were relatively evenly distributed between low and high levels. In *Critical Appraisal* and *Implementation of Applicable Evidence*, learning outcomes were primarily placed at low to intermediate taxonomy levels.

Example learning outcomes:

- *Has knowledge of the ageing process and the distinctive needs of older adults* (HEI 7). Mapped to: Low level, cognitive domain.
- *Is able to practice evidence-based nursing* (HEI 11). Mapped to: Intermediate level, psychomotor domain.
- *Is able to map deficits in the patient’s self-care resources and propose appropriate measures to alleviate discomfort and compensate for self-care deficits* (HEI 6). Mapped to: High level, psychomotor domain.

### The main competence area ‘professional nursing process’

A total of 36 instances of learning outcomes related to the main competence area *Professional Nursing Process* were identified. The sub competence area *Professional Critical Thinking* was most widely represented across institutions, followed by *Specific Critical Thinking*, whilst *General Critical Thinking* was included at relatively few higher education institutions.

Across sub competence areas, learning outcomes were primarily placed at the intermediate taxonomy level. *General Critical Thinking* was limited to the intermediate level and *Specific Critical Thinking* was mainly at the intermediate level, with few learning outcomes at the low level and none at the high level. *Professional Critical Thinking* spanned intermediate to high levels, although high-level learning outcomes were limited in number.

Example learning outcomes:

- *Has knowledge of basic systematic clinical examination and assessment *(HEI 13). Mapped to: Low level, cognitive domain.
- *Critically reflects on one’s own and others’ nursing practice based on relevant knowledge* (HEI 8). Mapped to: Intermediate level, affective domain.
- *Is able to apply professional knowledge of health and illness to systematically observe, assess, decide, implement and document appropriate nursing interventions for older and care-dependent individuals, as well as to evaluate their effects and adjust them as needed* (HEI 1). Mapped to: High level, psychomotor domain.

### The main competence area ‘nursing administration’

A total of 33 instances of learning outcomes related to the main competence area *Nursing Administration* were identified. Learning outcomes were predominantly associated with the sub competence area *Management and Leadership*, whilst *Informatics and Documentation* was somewhat less well represented across institutions*.*

Within both *Management and Leadership* and *Informatics and Documentation*, taxonomy levels were largely distributed between low and intermediate levels, with no learning outcomes categorised at the high taxonomy level.

Example learning outcomes:

- *Is familiar with quality indicators and standard terminology in the documentation of nursing* (HEI 3). Mapped to: Low level, cognitive domain.
- *Is able to document nursing care in accordance with applicable legislation and requirements for professional responsibility* (HEI 12). Mapped to: Intermediate level, psychomotor domain.

### The main competence area ‘work readiness and professional development’

A total of 57 instances of learning outcomes related to the main competence area *Work Readiness and Professional Development* were identified. The sub competence areas *Safety Procedural Skills* and *Personal Characteristics* were most widely represented across institutions, whilst *Legality*, *Preventative Health Services* and *Mentoring Competency* were less well represented, with *Mentoring Competency* appearing only once.

Taxonomy levels within this main competence area were predominantly intermediate. *Safety Procedural Skills* and *Personal Characteristics* were mainly placed at the intermediate level, with a limited number of high-level learning outcomes within *Safety Procedural Skills*. *Legality* and *Preventative Health Services* were primarily represented at low to intermediate levels, and high-level learning outcomes were scarce overall.

Example learning outcomes:

- *Has the capacity for self-insight, seeks guidance, and works on personal and professional development* (HEI 10). Mapped to: Intermediate level, affective domain.
- *Is able to plan and implement targeted measures to reduce adverse events and variation in healthcare services* (HEI 12). Mapped to: Intermediate level, psychomotor domain.
- *Is able, under supervision, to plan and carry out safe medication management, including drug calculation, and contribute to quality assurance of the individual patient’s medication use* (HEI 13). Mapped to: High level, psychomotor domain.

In sum, all five main competence areas are present in the learning outcomes. *Individualised Care* is the most extensively expressed, particularly through outcomes related to *Value and Ethical Codes* and *Therapeutic Communication*, which span multiple taxonomic levels. In contrast, the sub competence areas *Participatory Decision-Making* and *Cultural Humility* occur far less frequently. *Evidence‑Based Nursing Care* is dominated by learning outcomes related to *Knowledge Acquisition*, whilst learning outcomes at the sub competence area *Critical Appraisal and Implementation of Applicable Evidence* are less common. The learning outcomes span all taxonomic levels. In *Professional Nursing Process*, most learning outcomes are situated at intermediate taxonomic levels, with higher-level formulations being relatively rare and occurring only within *Professional Critical Thinking*. In *Nursing Administration*, the learning outcomes are limited to lower and intermediate levels, and the sub competence area *Informatics and Documentation* has the least representation. Within *Work Readiness and Professional Development*, *Safety Procedural Skills* and *Personal Characteristics* are most frequently represented. In this main competence area, the learning outcomes are primarily placed at intermediate taxonomic levels, with some exceptions.

Overall, the learning outcomes articulated in subject plans for first-year nursing home placements reveal consistent educational priorities, with a strong emphasis on competencies formulated at intermediate and lower taxonomic levels and limited presence of high‑level expectations across institutions.

## Discussion

This study aimed to identify the professional competence areas articulated in learning outcomes for first-year nursing students’ nursing home placements and to examine how these outcomes are distributed across cognitive, affective and psychomotor domains and their associated taxonomic levels. The results reveal a clear pattern in how learning outcomes for first-year nursing home placements are formulated. All five competence areas were represented; however, the distribution was uneven, and learning outcomes were predominantly positioned at low and intermediate taxonomic levels.

Learning outcomes are concentrated within a limited set of competence areas and are positioned predominantly at intermediate and, to some extent, low taxonomic levels. These patterns reflect underlying educational priorities that shape how first-year students are positioned as learners in nursing home placements.

The strong emphasis on *Individualised Care*, particularly through learning outcomes related to ethical awareness and communication, suggests that higher education institutions regard relational and ethical competencies as the foundation for safe participation in care. Although this prioritisation is pedagogically sound, the predominance of low and intermediate taxonomic levels indicates that students are largely expected to recognise and apply core relational behaviours rather than engage with more advanced interpretive or evaluative processes. This aligns with the finding that sub competence areas such as *Participatory Decision-Making* and *Cultural Humility* appear infrequently and are not formulated at higher taxonomic levels. Their limited presence may reduce opportunities for students to begin developing the ability to navigate the nuanced interpersonal and contextual dimensions of individualised care [34].

Consequently, when learning outcomes primarily emphasise recognition and basic understanding, as well as the simple description and application of these dimensions, students may have fewer opportunities to begin developing higher-level clinical reasoning [20]. This includes critical interpretation, synthesising complex information and making independent evaluative judgements, as well as assessing conflicting demands arising from patient preferences, risk considerations and resource constraints. Consequently, care practices may appear respectful and well-intentioned yet fall short of being fully person-centred when clinical situations require complex, value-sensitive decision-making [22].

Within *Evidence-Based Nursing Care*, the predominance of learning outcomes focused on knowledge acquisition reflects an appropriate early curricular priority. However, learning outcomes situated at higher taxonomic levels, particularly those involving appraisal or interpretation of evidence, are relatively rare. While sophisticated evidence appraisal is neither expected nor realistic for first-year students, the limited emphasis on inquiry-oriented learning outcomes may nevertheless delay the development of analytical habits that are essential for later clinical reasoning [6, 28].

The uneven representation of *Professional Nursing Process* further underscores this issue. *General Critical Thinking* and *Specific Critical Thinking* are rarely formulated and not at high taxonomic levels, in contrast to *Professional Critical Thinking*, which is represented across all higher education institutions and at higher taxonomic levels. This suggests that critical thinking is often conceptualised as procedural compliance rather than a broader cognitive disposition [6]. This risks reinforcing a task‑based orientation to practice rather than fostering engagement with complexity and uncertainty, which is an essential component of judgement in dynamic nursing home settings [28]. Limited articulation of general and higher‑order critical thinking may therefore restrict the early development of clinical judgement [19].

*Nursing Administration* is primarily represented through learning outcomes placed at low taxonomic levels, which reflects a focus on operational skills rather than analytical or evaluative ones. Although early-stage students should not be expected to assume leadership responsibilities, the absence of higher-level expectations relating to prioritisation, coordination or evaluation may inadvertently signal that these dimensions are peripheral rather than foundational elements of nursing practice [38].

The competence area *Work Readiness and Professional Development* is particularly represented through learning outcomes related to *Safety Procedural Skills* and *Personal Characteristics*. These are appropriately situated at intermediate levels, reflecting expectations of applied competence and professional behaviour. Nevertheless, the predominance of intermediate-level learning outcomes raises questions about the extent to which early development of judgement and responsibility is encouraged. Furthermore, the near absence of learning outcomes related to mentoring competency suggests that students are rarely positioned as active contributors to the learning of others [38]. This absence may position students primarily as passive recipients of supervision rather than as contributors to others’ learning [19]. By contrast, early exposure to mentoring roles can support professional identity development and foster a sense of collective responsibility within nursing practice [38].

The findings should also be considered in light of the qualification framework context within which learning outcomes are formulated. Frameworks differ in how professional competence is conceptualised and described, with some placing greater emphasis on integrated performance and others distinguishing more explicitly between knowledge, skills and competence. Such differences may influence how learning outcomes are interpreted and operationalised in educational and clinical contexts, which may affect comparability across institutions and countries. Future research could explore how different qualification framework traditions shape the translation of learning outcomes into practice expectations and assessment criteria in health professions education.

## Conclusion

This study provides new insight into how professional competencies for first-year nursing students are articulated in learning outcomes for clinical placements in nursing homes. Across the 13 higher education institutions, the learning outcomes reflect all five main competence areas in the chosen framework. However, the distribution shows a clear predominance of learning outcomes related to individualised care and a consistent emphasis on intermediate and low taxonomic levels across domains. Although these formulations likely reflect an intention to support safe participation and the development of basic professional practice, they also limit the expected level of analytical engagement and the overall analytical depth required of novice students. Competence areas that require value-sensitive reasoning, cultural humility, evidence appraisal and broader critical thinking were less frequently represented or seldom articulated at high taxonomic levels. Consequently, students may enter their first clinical placement with expectations that encourage correct behaviour and task-oriented participation rather than early development of reflective judgement and context-dependent decision-making.

### Implications for education and curriculum development

The findings highlight a need to review how learning outcomes for early clinical placements in nursing homes are structured and taxonomically positioned. Strengthening higher-level expectations, such as analytical assessment, evidence use and shared decision making, would better prepare students for the complexity of nursing home practice, while still balancing foundational relational skills with inquiry-oriented learning.

Such revisions should not be interpreted as implying that first-year students are expected to achieve mastery of advanced competencies. Rather, the aim is to support a more deliberate taxonomic progression in learning outcomes, whereby foundational competencies are combined with opportunities for guided development of analytical, reflective and reasoning skills. This may better support students’ gradual progression towards higher levels of competence in later clinical placements.

The variation across higher education institutions underscores the importance of greater coherence in competence expectations. Programme leaders and course coordinators may review first-year nursing home placement plans to identify underrepresented competence areas and taxonomic levels. Targeted revision of selected learning outcomes to incorporate more analytically demanding expectations, such as basic evidence appraisal, value-sensitive reasoning and shared decision-making, may be feasible without extensive curriculum redesign. Clinical mentors and practice coordinators can also contribute by supporting higher-order learning through structured reflection, case discussions and assessment practices aligned with learning outcomes.

Shared principles for taxonomic progression and clearer, more ambitious learning outcomes can enhance comparability and better support mentors in fostering genuine professional development rather than primarily task-oriented participation.

### Implications for future research

Future research should explore how the formulation of learning outcomes influences students’ actual learning experiences, professional identity formation and development of clinical reasoning during early clinical placements. Comparative studies across different clinical contexts may reveal whether similar patterns of emphasis and taxonomic level appear elsewhere in the curriculum. Studies examining how mentors and students interpret and apply learning outcomes in practice may also offer important insights into how such formulations shape pedagogical practice and professional expectations in real clinical environments.

## Supplementary Information

Supplementary Material 1.

## Data Availability

The data analysed during the current study are not publicly available, as they consist of institutional curriculum documents obtained with permission and used for analytical purposes. Aggregated data supporting the findings are included in the article and supplementary material. Further information is available from the corresponding author upon reasonable request.
