# Supplementary material for: Expected professional competencies articulated in learning outcomes for novice nursing students in early nursing home placements: a document analysis
Source: BMC Med Educ. 2026 Jul 30;26:1730. doi: 10.1186/s12909-026-10048-6 (PMC13711753; doi:10.1186/s12909-026-10048-6)
Supplement: Supplementary file 1 [file 12909_2026_10048_MOESM1_ESM.docx]

| **Illustrative Learning Outcomes** | **Categorisation into**  **Main Competence Area;**  **Sub Competence Area** | **Interpretation** | **Mapping** | |
| --- | --- | --- | --- | --- |
|  |  |  | **Domain of Learning** | **Taxonomic Levels** |
| *Is familiar with ethical and legal challenges associated with the use of technology and digital solutions* (HEI 8) | Individualised care; Value and ethical codes | Recalls previous learned information. | Cognitive Domain:  **Remember** | Low |
| *Is familiar with how acute and chronic illnesses and conditions affect patients and their next of kin* (HEI 10) | Evidence-based nursing care; Knowledge acquisition | Comprehends the meaning and interpretation of instructions and problems. | Cognitive Domain:  **Understand** | Low |
| *Is able to organise and carry out their own work in an appropriate and professionally sound manner* (HEI 12) | Nursing administration; Management and leadership | A state of preparatory adjustment or readiness for a specific action or experience. | Psychomotor Domain:  **Set** | Low |
| *Can apply knowledge of professional ethical guidelines and applicable legislation in their practice* (HEI 2) | Individualised care; Value and ethical codes | Applies what was learned in the classroom into novel  situations in the workplace. | Cognitive Domain:  **Apply** | Intermediate |
| *Applies relevant professional and academic modes of expression, both in writing and orally* (HEI 5) | Evidence-based nursing care; Knowledge acquisition | An early stage in learning a complex skill that includes imitation and trial and error. | Psychomotor Domain:  **Guided Response** | Intermediate |
| *Is able to observe risk factors and apply knowledge of prevention and health promotion* (HEI 10) | Professional nursing process; General critical thinking | Assesses specific values and phenomena, expressed through the student’s observable behaviour. | Affective Domain:  **Valuing** | Intermediate |
| *Develops a plan for nursing care based on the patient’s needs* (HEI 8) | Professional nursing process; Specific critical thinking | Learned responses have become habitual and the movements can be performed with some confidence. | Psychomotor Domain:  **Mechanism** | Intermediate |
| *Performs basic nursing skills with accuracy and fluency, and maintains the aesthetic aspect of nursing practice* (HEI 9) | Evidence-based nursing care; Knowledge acquisition | Skilfully performs motor acts that involve complex movement patterns. Proficiency is indicated by quick, accurate and highly coordinated performance. | Psychomotor Domain:  **Complex Overt Response** | High |
| *Applies ethical principles, professional ethical guidelines and relevant legislation that reflect professional, responsible and safe nursing practice* (HEI 4) | Individualised care; Value and ethical codes | Integrates professional ethical principles and legal requirements into coherent professional judgment, demonstrates the ability to prioritise and act consistently in ethically complex situations. | Affective Domain:  **Organising** | High |
| *Is able to use technology and digital solutions in clinical studies in nursing homes to support the resources of patients and next of kin, safeguard their right to participation and contribute to their experience of coping* (HEI 13) | Individualised care; Participatory decision-making | Skills are well developed and the student can modify movement patterns to fit special requirements. | Psychomotor Domain: **Adaptation** | High |

HEI = higher education institution. Institutions are anonymised and labelled HEI 1–HEI 13
